# Mesoporous Materials: From Synthesis to Applications

**DOI:** 10.3390/ijms20133213

**Published:** 2019-06-30

**Authors:** Juan Antonio Cecilia, Ramón Moreno Tost, María Retuerto Millán

**Affiliations:** 1 Department of Inorganic Chemistry, Crystallography and Mineralogy, University of Málaga, 29071 Málaga, Spain; 2 Instituto de Catálisis y Petroleoquímica, C/Marie Curie 2, Cantoblanco, 28049 Madrid, Spain

Mesoporous silica are inorganic materials, which are formed by the condensation of sodium silicate or silicon alkoxides around an ordered surfactant used as template. The synthesis of these porous silica, as well as the porous structure depends on several parameters such as silicon source, the morphology of the surfactant, pH conditions, ionic strength, aging temperature, aging time, and so on. These synthetic conditions directly affect the structure, specific surface area, pore size, pore volume or wall thickness.

Mesoporous silica had a great impact in the 90s, when the Mobil company designed the mobil crystalline material (MCM) with different morphological characteristics in 1992 [1]. Thus, these authors synthesized MCM-41, MCM-48 and MCM-50 mesophases, which showed hexagonal, cubic and lamellar mesostructures, respectively [1]. In all cases, the porous silica were synthesized using alkyl ammonium salts, as the structure directing agent, and sodium silicate or tetraethylorthosilicate (TEOS) in basic medium as the silicon source [1]. In all cases, the pore size did not exceed 4.0 nm and the wall thickness was below 2.0 nm.

In 1998, an ordered mesoporous material, denoted as Santa Barbara (SBA) mesoporous molecular sieve, was firstly reported by Zhao et al. [2]. SBA-15 with hexagonal ordering and SBA-16 with lamellar structure were widely studied in later years. SBA are synthesized using non-ionic surfactants derived of poly (propylene oxide) and poly (ethylene oxide), as pore directing agents in acidic medium. The main advantage of these porous silica in comparison to MCM is related to the larger pore thickness, which provides them with greater thermochemical stability. In addition, the pore size of SBA can be modulated from 2 to 50 nm, expanding the number of applications. In the same way, a group at the Michigan State University, synthesized other porous silicas denoted as MSU, using non-ionic surfactants at neutral pH, obtaining a pore diameter between 2.1–8.0 nm and a wall thickness of 1.5–4.0 nm [3]; however, MSUs display less organized structures than the previous mentioned materials [1,2,3].

Since the 90s, the pore size has been modulated as a function of the application. Thus, the textural properties can be modified by controlling the synthetic conditions (aging time and temperature), by the insertion of swelling organic molecules (aliphatic hydrocarbons, aromatics, alkylamines), by adjusting of template, by the insertion of heteroatoms in the porous silica framework or by changing the removal conditions of the template [4,5,6].

Taking into account the versatility of the porous silica, some of the applications are explored below.

## 1. Immobilization of Bioactive Molecules and Dyes

The modulation of the pore size in mesoporous silica allow them to host bulky molecules such as drugs or enzymes as well as other organic compounds such as pigments and dyes. In all cases, the adsorption processes generally take place through adsorption, covalent binding, cross-linking, encapsulation and entrapment [7,8,9]. On the one hand, the immobilization depends on the dimensions of the biomolecule, its hydrophobic/hydrophilic nature or the superficial charge, and on the pore diameter of the porous silica [10]. Generally, the immobilization of these organic compounds is favored in porous silica with high surface area, and the pore diameter must be slightly higher than the diameter of the adsorbate. On the other hand, the existence of silanol groups on the surface of the mesoporous silica can favor the immobilization of the biomolecule or any organic compound by electrostatic interactions. In the same way, the silanol groups can be functionalized to modify the superficial chemical of the mesoporous silica on the surface, incorporating a wide range of functional groups such as –NH_2_, –SH, or –COOH, which can enhance immobilization capacity of the organic molecules [11,12]. However, the incorporation of these functional groups can also affect the mesostructure of the porous silica, narrowing the dimensions of meso-channels. In some cases, the incorporation of heteroatoms on the surface of the porous silica modify the physicochemical properties of the mesoporous silica, enhancing the acidity or basicity of the adsorbent [13,14]. This fact supposes an improvement of the adsorption capacity, although it also leads to a stronger adsorbent–adsorbate interaction in such a way that desorption is more difficult [13].

## 2. Mesoporous Silica as Catalyst and/or Support

In recent years, the mesoporous silica have been considered one of the most promising materials in the field of catalysts due to the ease of tailoring the properties of the catalytic processes [1,2,3]. Mesoporous silica are hardly used as a catalyst themselves; however, the incorporation of some heteroatoms can modify the slightly acidic character of silica. Thus, the incorporation of some heteroatoms such as Al, Zr, Ti, Nb or Ta in the synthetic step or in post-synthesis can provide acid sites to the porous silica [15,16]. The incorporation of these heteroatoms must be modulate since the incorporation of a high proportion of these heteroatoms in the synthetic step can affect the condensation of the porous silica, leading to more disordered structures with lower specific surface area. In the same way, the addition of a high proportion of these heteroatoms after the formation of the porous silica can also affect their textural properties since these heteroatoms can partially block the porous structures in such a way that a large proportion of the pores are not available. In the same way, the incorporation of alkaline and especially alkaline earth, can provide basic sites to these porous materials, expanding the range of reactions of these mesoporous silica [17].

Mesoporous silica also have a great potential to disperse small metallic or metal oxide particles on the surface as well as inside the porous structure [18]. This fact supposes an increase of the available active sites, which are necessary in acid, basic, hydrogenating or oxidizing reactions.

## 3. Polymer Reinforcement

Mesoporous silica also have great potential as additives to improve the tribological, chemical and thermomechanical properties of polymer-based materials (PBMs) [19,20]. The high porosity of the mesoporous silica allows them to host a high amount of polymer chains. Accordingly, several composites have been reported such as poly(ethylene oxide)/MCM-41, styrene butadiene rubber/MCM-41 [21]. Generally, the obtained composites present a higher elastic modulus at every evaluated temperature indicating higher polymer chain stiffness.

Other authors have reported that the mesoporous silicas can host monomers and then the polymerization in situ inside the mesoporous can take place. This methodology was followed by the synthesis of several polymers such as polymethyl methacrylate, polystyrene and polybutyl acrylate on porous silica and polymers with higher toughness, when the intrapore polymer was flexible, were obtained [19,20].

## 4. Porous Silica as Hard Template

The porous structure of the mesoporous silica can be used as template to synthesize carbon structures with well-defined morphologies such as a function of the 3D structure of the mesoporous silica. Ryo et al. performed the first research that synthesized ordered porous carbons, denoted as CMK-1, from MCM-48 impregnated with sucrose. Although, other templates have also been frequently used such as MCM-41, SBA-15, MSU or MCF [22,23,24]. The modulation of the pore size in the porous silica as a function of the catalytic condition will be a key factor in obtaining highly porous carbonaceous structures with interesting application perspectives.

Generally, the porous structure is impregnated with a carbon source such as sucrose, furfuryl alcohol, propylene or organic resin. Then, the composite is pyrolyzed to obtain a carbon matrix. As a next step, the siliceous structure is removed by leaching, using strong conditions (HF or NaOH) [23]. An alternative to reduce the cost of the synthesis of these carbonaceous structures is the use of sodium silicate which is a more economical source of silicon [25].
